# The Role of Gene Elongation in the Evolution of Histidine Biosynthetic Genes

**DOI:** 10.3390/microorganisms8050732

**Published:** 2020-05-13

**Authors:** Sara Del Duca, Sofia Chioccioli, Alberto Vassallo, Lara Mitia Castronovo, Renato Fani

**Affiliations:** Department of Biology, University of Florence, 50019 Sesto Fiorentino, Italy; sara.delduca@unifi.it (S.D.D.); sofia.chioccioli@unifi.it (S.C.); alberto.vassallo@unifi.it (A.V.); laramitia.castronovo@unifi.it (L.M.C.)

**Keywords:** gene elongation, histidine biosynthesis, patchwork hypothesis

## Abstract

Gene elongation is a molecular mechanism consisting of an in-tandem duplication of a gene and divergence and fusion of the two copies, resulting in a gene constituted by two divergent paralogous modules. The aim of this work was to evaluate the importance of gene elongation in the evolution of histidine biosynthetic genes and to propose a possible evolutionary model for some of them. Concerning the genes *hisA* and *hisF*, which code for two homologous (β/α)_8_-barrels, it has been proposed that the two extant genes could be the result of a cascade of gene elongation/domain shuffling events starting from an ancestor gene coding for just one (β/α) module. A gene elongation event has also been proposed for the evolution of *hisB* and *hisD*; structural analyses revealed the possibility of an early elongation event, resulting in the repetition of modules. Furthermore, it is quite possible that the gene elongations responsible for the evolution of the four proteins occurred before the earliest phylogenetic divergence. In conclusion, gene elongation events seem to have played a crucial role in the evolution of the histidine biosynthetic pathway, and they may have shaped the structures of many genes during the first steps of their evolution.

## 1. Introduction

In the study of molecular evolution, because there are many missing links between the past and the present, the investigation of extant structures is fundamental to attempts to infer the ancestral situation [1]. The ever-increasing availability of complete genome nucleotide sequences from different organisms provides a huge dataset for the study of the structure and organization of genes and genomes; this makes possible a better understanding of the molecular mechanisms responsible for the evolution of metabolic pathways [2]. It is generally accepted that ancestral protein-coding genes were probably short sequences, encoding little polypeptides presumably corresponding to functional and/or structural domains. The evolution of genes, comprising the increase of their size and complexity, is the result of different molecular mechanisms including (i) gene duplication, (ii) gene fusion, and (iii) gene elongation [3].

### 1.1. Gene Duplication and Gene Fusion Events

The duplication of a gene can provide benefits to the organism simply because extra amounts of protein or RNA products are provided; however, one of the most important outcomes of gene duplication is the emergence of novel functions [4]. The duplication of genes and their subsequent functional divergence can lead to the formation of paralogous genes families that are evolutionarily related but have different functions [5]. In fact, after a gene duplicates, one of the two copies becomes dispensable and can undergo (several types of) mutational events. Over time, the mutation-containing gene can become a (pseudo)gene that can either be deleted from the genome or acquire a novel function with respect to the parental gene [4]. Hence, gene duplication is one of the principal mechanisms involved in protein evolution [6].

In addition to gene duplication, one of the major forces driving gene evolution is the fusion of independent cistrons leading to bi- or multifunctional proteins [3]. Gene fusions allow the physical association of different protein domains (catalytic or regulatory) and can occur between genes located near to each other or far apart on the DNA molecule(s). Fusions frequently involve genes coding for proteins that function in a concerted manner, such as successive enzymes in metabolic pathways, enzymes and their regulatory domains, or DNA- and ligand-binding domains in prokaryotic transcriptional regulators [7].

### 1.2. The Gene Elongation Mechanism

**Figure 1:**
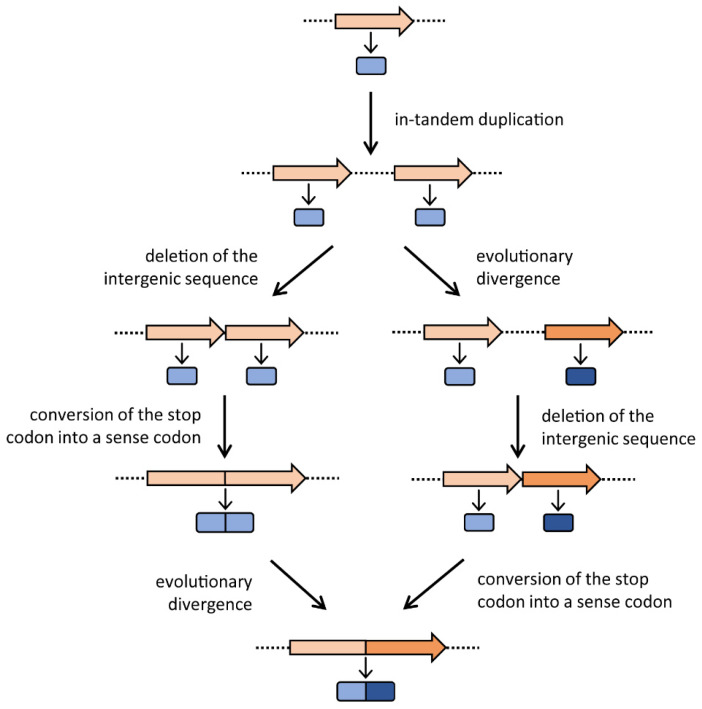
The gene elongation mechanism. The two possible evolutionary routes are depicted. Genes are represented with arrows, and the encoded proteins with rounded rectangles. Representation adapted from Fani and Fondi [3].

Gene elongation consists of an in-tandem gene duplication, which produces two (or more) copies of the same gene, followed by the loss of the intergenic region and the mutation of the stop codon of the first copy into a sense codon, resulting in the elongation, by fusion, of the initial gene and its copy. Thus, gene elongation is actually the combination of gene duplication and gene fusion. The newly formed gene is constituted by two paralogous modules, which might independently undergo different mutations and further duplications [3] that, over time, can cover up the traces of these early events. As shown in Figure 1, once the two copies have originated, in principle they might follow two different evolutionary pathways in which they can either immediately fuse and then diverge or, vice versa, diverge and then fuse. The final outcome of these two alternative pathways is the same: the formation of a gene constituted by two divergent paralogous modules. Gene elongation produces two or more copies of the same protein fold within a single polypeptide chain, often generating a direct repeat of domains and thus a pseudo-symmetrical structure with internal symmetry axes [1]. Many present-day proteins show internal repeats of amino acid sequences, which often correspond to functional or structural domains; since gene elongation has occurred in so many cases, this event must be considered an evolutionary advantage. The biological significance of proteins with repetitive structures might include (i) the improvement of the protein function by increasing the number of active sites; (ii) the acquisition of an additional function by modifying a redundant segment [3], thus obtaining a bifunctional enzyme; and/or (iii) the stabilization of a protein structure, thus increasing the enzyme’s catalytic activity. Lots of cases of genes with internal sequence repetitions are reported in the literature. For example, in the bacterial ferredoxin, the second half of the amino acid sequence is an almost exact duplicate of the first [8]. Tang et al. [9] observed that the pepsin family of proteases have an intramolecular two-fold symmetry axis that relates two topologically similar domains, and proposed a mechanism for its evolution by gene elongation. The *carB* gene of *Escherichia coli*, which encodes a subunit of carbamoyl-phosphate synthetase, was proposed to be formed by the duplication of an ancestral gene, since its amino acid sequence shows a highly significant homology between the amino- and carboxyl-terminal halves of the protein [10]. Rubin et al. [11] found that the two halves of Gram-negative bacterial tetracycline efflux pumps share a process of tandem gene duplication and divergence. Gupta and Singh [12] suggested a model for the evolution of the heat-shock protein 70 (Hsp70) of Archaea and Bacteria based on gene duplication. Moreover, domain fusions have occurred in the evolution of the ATP binding cassette (ABC) superfamily [13]. However, the most documented case of gene elongation involves the histidine biosynthesis genes *hisA* and *hisF*; they are paralogous and may have originated from the duplication (and the subsequent divergence and fusion of the two resulting copies) of an ancestral gene, half the size of the current genes [14]. To the best of our knowledge, this is the only reported case of a “universal” gene elongation event; indeed, *hisA* and *hisF* genes share the same internal organization in all histidine-synthesizing organisms, suggesting the antiquity of this elongation event and that it occurred (long) before the appearance of the Last Universal Common Ancestor (LUCA) [15]. Thus, gene elongation might have shaped the structures of many genes during the first steps of molecular and cellular evolution.

### 1.3. Histidine Biosynthesis and Its Evolution

**Figure 2:**
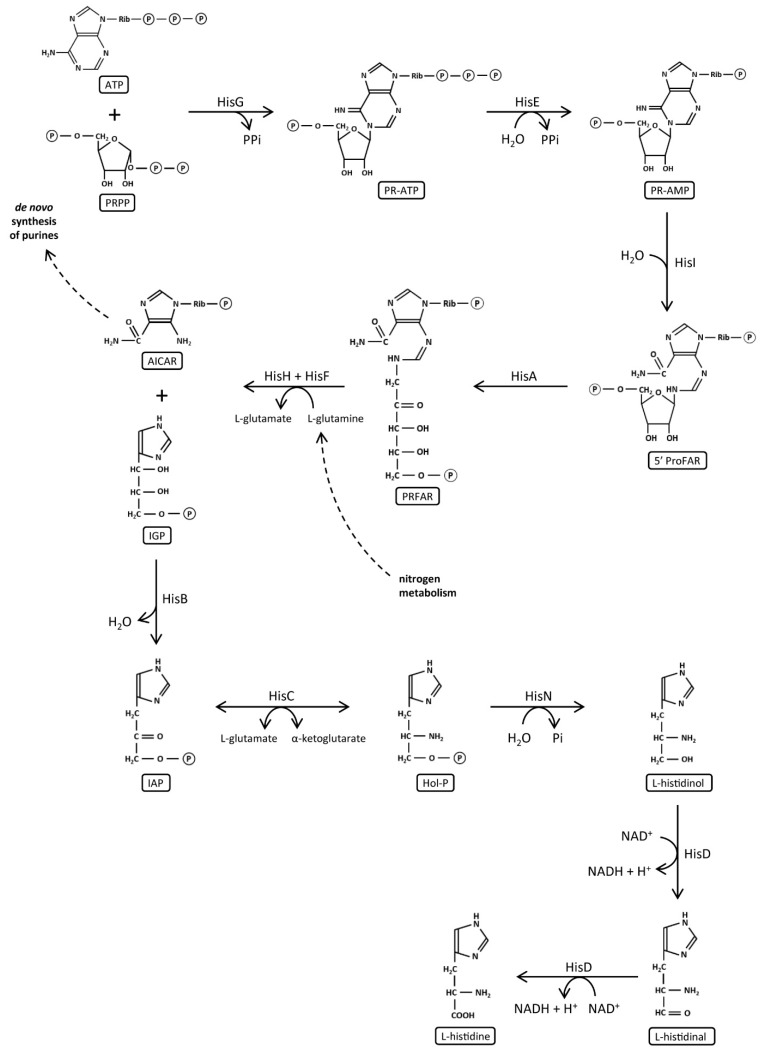
Biosynthetic pathway of L-histidine. Representation adapted from Kulis-Horn et al. [16] and Alifano et al. [17].

One of the most studied metabolic pathways, which shows a plethora of gene structures and organizations, is histidine biosynthesis, an ancient metabolic pathway present in Bacteria, Archaea, lower eukaryotes, and plants [16] that converts 5-phosphoribosyl-1-pyrophosphate (PRPP) to L-histidine through 10 enzymatic reactions (Figure 2). L-histidine biosynthesis plays an important role in cellular metabolism, being interconnected with both the de novo synthesis of purines and to nitrogen metabolism, which is why it is defined as a “metabolic cross-roads” [15]. It is one of the best characterized pathways from different biochemical, genetic, and evolutionary viewpoints. Its study is interesting because of (i) the presence of several quite uncommon reactions for a biosynthetic pathway, (ii) the existence of connections with other metabolic pathways, (iii) the structural features of several of the biosynthetic enzymes [17], and (iv) the different organization of *his* genes in different (micro)organisms. Genetic studies of this pathway have contributed, over the years, to the formulation of key concepts of molecular biology, like the operon hypothesis [18], and to the understanding of the mechanisms underlying the regulation of biosynthetic pathways, such as feedback inhibition, energy charge, and the setting of basal biosynthetic enzyme levels [19].

**Figure 3:**
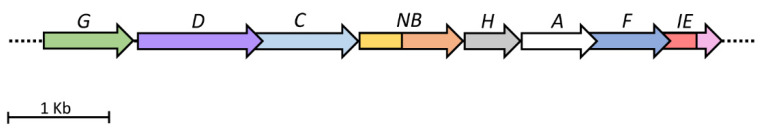
Schematic representation of *Escherichia coli his* operon.

This pathway has been extensively studied mainly in the enterobacteria *E. coli* and *Salmonella enterica*, for which a lot of information is available, including pathway structure, gene expression, and growing volumes of sequence data [15,19]. In these two bacteria the route is identical, consisting of nine intermediates and eight enzymes encoded, respectively, by eight genes arranged in a compact operon (*hisGDC[NB]HAF[IE]*) (Figure 3): three of these (i.e., *hisNB*, *hisD*, and *hisIE*) code for bifunctional enzymes, while another one is heterodimeric, being composed of the *hisH* and *hisF* gene products [17].

Chemical and biological evidence suggests that histidine was already present on Earth during the long period of chemical abiotic synthesis of organic compounds. It is known that the imidazole group of the lateral chain of His is present in the active sites of many enzymes, playing an important role in metabolism. Indeed, the dipeptide His–His was one of the first simple peptides to show the ability to form peptide bonds under plausible prebiotic conditions. It is reasonable to speculate that small peptides containing histidine might have been involved in the prebiotic formation of other peptides and nucleic acid molecules [20,21]. Since histidine is the only amino acid of which biosynthesis starts from a nucleotide rather than small metabolic intermediates, it has also been suggested that histidine may be the molecular vestige of a catalytic ribonucleotide from an earlier biochemical era in which RNA played a major role in catalysis [22,23]. If primitive enzymes required histidine, then the exhaustion of the prebiotic supply of histidine imposed a selective pressure favoring those organisms capable of synthesizing this molecule. The need to produce histidine suggests that this biosynthetic route is ancient and might have been assembled long before the appearance of LUCA [3,21]. Comparison of the *his* genes available in databases shows that after the divergence from LUCA, the structure, organization, and order of these genes underwent substantial rearrangements in the three cell lineages (Archaea, Bacteria, and Eukarya) [17]; indeed, a wide variety of different clustering strategies of *his* genes have been documented [3]. Although it is not possible from analysis of the available data to infer the organization and localization on the genome of the *his* genes in the last common ancestor, the same analysis may help to understand the primitive structures of some of them and their evolution [17]. Nowadays, this reconstruction of evolutionary dynamics is facilitated by the increasing number of complete genome sequences available. Analyses of the structure of *his* genes revealed that at least three different molecular mechanisms played an important role in shaping this pathway [2], i.e., gene duplication, gene fusion, and gene elongation, making this route a very good model for understanding the molecular mechanisms driving the shaping of metabolic pathways [15].

Since it is supposed that in many cases, functionally related genes arise from the duplication of an ancestral gene and subsequent evolutionary divergence [6], in the pre-genomic era, Horowitz [24] proposed that all of genes belonging to an operon might have been originated from an ancestral gene via duplication and divergence; as a corollary, all the histidine biosynthetic genes could have evolved via the duplication of a single ancestor gene. However, analysis of the nucleotide sequence of *his* genes in *E. coli* and *S. enterica* has not revealed any extensive sequence homology between these different genes [14]. In spite of this, an event of gene duplication led to the formation of two *his* genes, *hisA* and *hisF*, starting from a common ancestral gene. Gene fusion appears to have been one of the most important mechanisms of gene evolution in the histidine biosynthesis [25]; indeed, at least seven of the ten biosynthetic genes (i.e., *hisD*, *hisB*, *hisN*, *hisI*, *hisE*, *hisF*, and *hisH*) underwent single or multiple fusion events in different prokaryotic and eukaryotic phylogenetic lineages [15]. Finally, regarding the gene elongation event, the most documented example concerns the evolution of *hisA* and *hisF* genes, encoding two (β/α)_8_-barrel (TIM-barrel) proteins [15].

The aim of this work was to evaluate the importance of gene elongation in the evolution of histidine biosynthetic genes and, if possible, to refine the model depicted for the evolutionary history of some of them and/or to trace the evolutionary trajectories of other ones.

## 2. Materials and Methods 

### 2.1. Three-Dimensional Structure Prediction 

All the 3D protein structures reported in the present work were predicted using Phyre2 software [26], and were visualized and modified with UCSF Chimera [27]. Files of the predicted structures are provided in PDB format in Supplementary Material S1.

### 2.2. PDB Search for Protein Structures

For HisA/HisF, HisB (IGPD), and HisD (HDH), available in the Protein Data Bank (PDB) archive [28], the secondary and 3D structures were investigated. The available structures (as of April 2020) are reported below.

HisA PDB IDs:

- 1QO2, 2CFF, 2W79—*Thermotoga maritima*;
- 1VZW, 2VEP, 2X30, 5DN1—*Streptomyces coelicolor*;
- 2AGK—*Saccharomyces cerevisiae*;
- 2Y85, 2Y88, 2Y89, 3ZS4—*Mycobacterium tuberculosis*;
- 4AXK—*Corynebacterium efficiens*;
- 4GJ1—*Campylobacter jejuni*;
- 4TX9, 4U28—*Streptomyces sviceus*;
- 4W9T, 4X9S—*Streptomyces* sp.;
- 4WD0—*Paenarthrobacter aurescens*;
- 4X2R—*Actinomyces urogenitalis*;
- 5AB3, 5ABT, 5G4W, 5G5I, 5G1T, 5G1Y, 5G4E, 5G2H, 5G2I, 5G2W, 5AC6, 5AC7, 5AC8, 5AHF, 5A5W, 5AHE, 5AHI, 5L9F—*Salmonella enterica*;
- 5L6U—*Salmonella heidelberg*.

HisF PDB IDs:

- 1GPW, 1THF, 1VH7, 2A0N, 2WJZ, 3ZR4, 4EWN, 4FX7, 5TQL, 6VDG—*Thermotoga maritima*;
- 1H5Y—*Pyrobaculum aerophilum*;
- 1JVN, 1OX4, 1OX5, 1OX6—*Saccharomyces cerevisiae*;
- 1KA9—*Thermus thermophilus*.

IGPD PDB IDs:

- 1RHY—*Filobasidiella (Cryptococcus) neoformans*;
- 2AE8—*Staphylococcus aureus*;
- 2F1D, 4MU0, 4MU1, 4MU3, 4MU4, 4QNJ, 4QNK, 5ELW, 5EKW, 5EL9, 6EZJ—*Arabidopsis thaliana*;
- 4GQU, 4LOM, 4LPF, 5XDS, 5ZQN—*Mycobacterium tuberculosis*;
- 5DNL, 5DNX—*Pyrococcus furiosus*;
- 6EZM—*Saccharomyces cerevisiae*;
- 6FWH—*Acanthamoeba castellanii*.

HDH PDB IDs:

- 1K75, 1KAE, 1KAH, 1KAR—*Escherichia coli*;
- 4G07, 4G09—*Brucella suis*;
- 4GIC—*Methylococcus capsulatus*;
- 5VLB, 5VLC, 5VLD—*Medicago truncatula*;
- 6AN0—*Elizabethkingia anophelis*.

### 2.3. Amino Acid Sequence Alignment

Amino acid sequences from HisA/HisF, IGPD, and HDH, available in the PDB, were downloaded from UniProt [29], aligned using BioEdit [30] through the ClustalW tool [31], and the conservation of the secondary structure organization was evaluated.

A total of 81 IGPD amino acid sequences from Archaea, 359 from Bacteria, and 95 from Eukarya were downloaded from UniProt to give a total of 535 IGPD sequences, chosen from every principal taxonomic group. Sequences were aligned using BioEdit through the ClustalW tool (Supplementary Material S2), and the conservation of the (D/N)XHHXXE motif was investigated.

## 3. Results and Discussion

### 3.1. *hisA* and *hisF* Gene Elongation

The *hisA* gene codes for a N’-[(5′-phosphoribulosyl)-formimino]-5-aminoimidazole-4-carboxamide-ribonucleotide (5′-ProFAR) isomerase catalyzing the fourth step of the histidine biosynthetic pathway, and the *hisF* gene encodes a cyclase catalyzing the fifth step (Figure 2) [19]. HisF is part of a heterodimeric complex, an imidazole glycerol phosphate (IGP) synthase composed of the cyclase subunit HisF and the glutamine amidotransferase (GAT) subunit HisH. The HisF:HisH association is a stable 1:1 dimeric complex [17], which is a branch-point enzyme, since its two products IGP and AICAR (5-aminoimidazole-4-carboxamide ribonucleotide) are used in histidine biosynthesis and in the de novo synthesis of purines, respectively [32]. HisA, like TrpF, catalyzes an Amadori rearrangement, i.e., the irreversible isomerization of an aminoaldose to an aminoketose [33]. The same chemical rearrangement is responsible for the imidazole ring closure by HisF [34]. HisA and HisF share the ability to bind PRFAR (N9-[(59-phosphoribulosyl)-formimino]-5-aminoimidazole-4-carboxamide-ribonucleotide): HisA catalyzes the formation of PRFAR, and HisF catalyzes an ammonia-dependent reaction in which PRFAR is converted to IGP and AICAR. Thus, they act like phosphoribosyl anthranilate isomerase (TrpF) and indole-3-glycerol phosphate synthase (TrpC), which catalyze successive reactions in the tryptophan biosynthetic pathway [35]. Both TrpF and TrpC are homologs to the HisF/HisA pair; although a prebiotic synthesis of tryptophan has been reported, it is likely that this amino acid has occurred later in evolution. Therefore, the HisF/HisA pair is more ancient than the TrpF and TrpC proteins, antiquity that is in agreement with the key catalytic role played by histidine in many extant enzymes [34].

**Figure 4:**
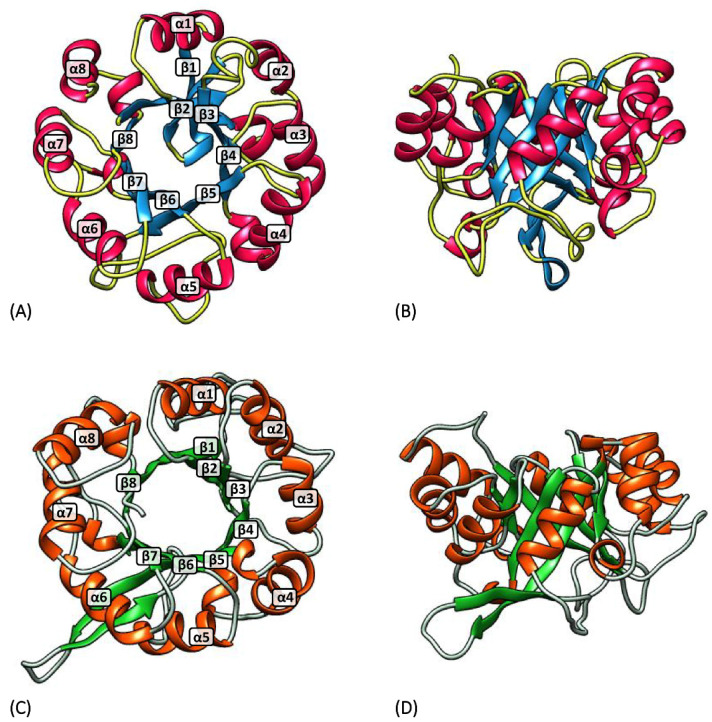
Three-dimensional structure of *E. coli* HisA (**A**,**B**) and HisF (**C**,**D**). (**A**,**C**) top view of the barrels; (**B**,**D**) side view. α-helices and β-strands are labeled.

The comparative analysis of the amino acid sequences of *hisA* and *hisF* gene products in different organisms showed a high degree of sequence similarity (ranging from 32% to 81%, considering similar amino acids), suggesting that the two genes are paralogous and that they originated from a common ancestor gene via duplication and subsequent evolutionary divergence [14]. HisA and HisF can be therefore considered examples of retrograde evolution of enzymes in a biosynthetic pathway; according to this hypothesis, when the substrate for an enzyme in a biosynthetic pathway is depleted, a new enzyme can evolve to supply the substrate via duplication of the original coding gene followed by divergent evolution [24,36]. A more elaborate analysis highlighted an internal duplication in both *hisA* and *hisF* genes. As proposed by Fani et al. [14], *hisA* and *hisF* may have originated from the duplication of a smaller ancestral gene, half the size of the current genes, followed by evolutionary divergence and fusion of the two derivatives. They proposed a “two-step model” for the evolution of *hisA* and *hisF* genes; according to this hypothesis, *hisA* originated from the duplication of a *hisA1* module, and *hisF* then originated from the duplication of *hisA*. The biological significance of this gene elongation event became clear when the structures of the HisA and HisF proteins were determined. Crystallographic studies showed that these two enzymes are structurally homologous (β/α)_8_-barrels (Figure 4) [37], with a two-fold repeat pattern; the first and second (β/α)_4_-half barrels of both enzymes are related by a two-fold axis of symmetry. These data led to the proposal of a model for the evolution of the HisA and HisF (β/α)_8_-barrels via two successive gene duplication events: a first duplication of a single gene encoding a (β/α)_4_-half barrel, subsequent fusion and modest divergent evolution, followed by a second gene duplication and diversification leading to the extant enzymes HisA and HisF [32]. The hypothesis that the *hisF* gene originated from *hisA* finds support in the organization and structure of these genes in different organisms; however, the possibility that *hisF* gave rise to *hisA* cannot be ruled out. Since the structure of *hisA* is the same in all the microorganisms in which this gene has been identified, it is likely that the duplication event leading to the entire *hisA* gene occurred before the earliest phylogenetic divergence [25]. Concerning the gene elongation event leading to the gene encoding the entire barrel, it is still not possible on the basis of the available data to discern between two different scenarios, i.e., the fusion event between the two copies could have occurred either before or after they underwent an evolutionary divergence.

**Figure 5:**
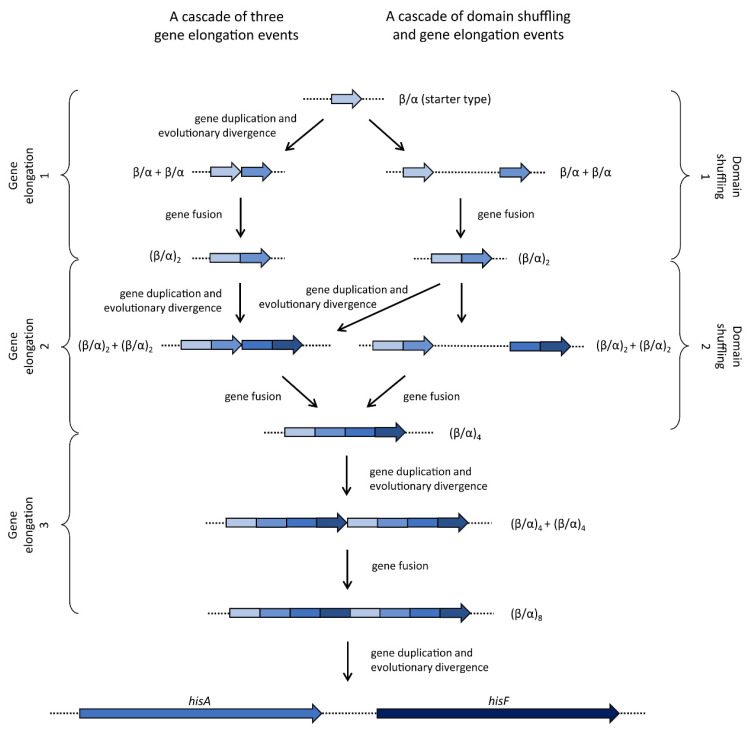
Evolutionary model proposed to explain the origin and evolution of *hisA* and *hisF* genes.

It is known that the (β/α)-barrel fold is a versatile structure for different enzymatic functions; indeed, 10% of known enzymes possess this fold [38], and catalyze very different reactions. The first protein that was discovered to have an eight-stranded (β/α)-barrel domain was triose phosphate isomerase, hence the name “TIM-barrel” [39]. The barrel structure is composed of eight concatenated (β-strand)-loop-(α-helix) units. The β-strands delimit the internal lumen of the barrel, whereas the α-helices form the external surface [15]. Although the fold is known as a barrel, the (β/α) structure is usually not circular in cross section, but rather elliptical [39]. The active sites are located at the C-terminal ends of the barrels, with the active site functional groups found in the loops that connect the β-sheets with the α-helices [38]. A large number of (β/α)_8_-barrel proteins (including HisA and HisF) contain phosphate-binding motifs formed by the C-terminal end of the seventh β-strand and the N-terminal end of the eighth α-helix in the barrel [35]. Considering that the (β/α)_8_-barrel fold is the most common active site scaffold, and that this fold may also represent an ideal scaffold for the creation of new enzyme activities, since the chemistry catalyzed by the protein can be altered without loss of specificity [40], much attention has been devoted to understanding the phylogeny of proteins sharing this fold [35]. During the first years of the 2000s, lots of experiments were performed to better understand the evolution of this class of proteins. Jürgens et al. [33], using random mutagenesis and selection, generated several HisA variants that catalyze the same reaction of TrpF both in vivo and in vitro, suggesting that HisA and TrpF may have evolved from one common ancestral enzyme of broader substrate specificity. This is in full agreement with the patchwork hypothesis of the evolution of metabolic pathways, independently proposed by Yčas [41] and Jensen [42], in 1974 and 1976, respectively. Noteworthy, this occurrence is observed in the actinomycetes *Streptomyces coelicolor* and *Mycobacterium tuberculosis*, where a single enzyme takes part in both histidine and tryptophan biosynthesis. Indeed, *trpF* is not present in their genomes, and the corresponding enzymatic activity is encoded by *priA*, the ortholog gene of *hisA* [43]. Gerlt and Babbitt [35] studied the evolution of IGP synthase. The N- and C-terminal (β/α)_4_-half barrels of HisF were separately expressed and purified. Each one assumed a stable and soluble homodimeric structure, but neither was catalytically active; in contrast, when expressed together, a functional heterodimer was formed, suggesting that the ancestral half-barrel gave a functional enzyme by homodimerization. Fani et al. [15] investigated the possibility of an even older gene elongation event involving (β/α)-mers smaller than the (β/α)_4_ units of the ancestral half-barrel precursor. We cannot exclude the possibility that an ancestral gene encoding a single (β/α)-module might represent one of the “starter types”, i.e., one of the few most ancestral genes not derived from the duplication of a pre-existing one [44]. According to this idea, that module might have been able to aggregate in a homo-octamer to form a still unstable and thus not efficient complete TIM barrel, which, according to the patchwork hypothesis [42], might have been embedded with a broad specificity. Subsequently, a “cascade” of three gene elongation events would have given rise to the complete ancestor of the extant TIM-barrel coding genes (Figure 5). These studies highlighted that the elongation event leading to the ancestor of *hisA* and *hisF* genes resulted in the covalent fusion of two half-barrels; once assembled, the complete gene underwent gene duplication, leading to the ancestor of *hisA*, *hisF*, and maybe all the TIM-barrel-protein-coding genes. This is the most parsimonious scenario to explain the origin and evolution of *hisA* and *hisF* genes. However, since, according to Woese [45], the early cells might have been embedded with multiple short informational molecules, it could be also possible that different copies of the (β/α) coding starter type might exist in the same or different DNA molecules in the progenote. Hence, the possibility that the ancestor (β/α)_2_ coding gene might have been the result of a domain shuffling event cannot be excluded a priori [46]. Once the two different (β/α) coding genes fused, the resulting gene might, in turn, have undergone a gene elongation event or one or more additional domain shuffling event(s) (Figure 5). Indeed, the degree of sequence similarity shared by the single (β/α) modules is not sufficient to support the three gene elongations model. However, the degree of sequence similarity between the two halves of the HisA and HisF proteins strongly suggests that the final molecular rearrangement leading to the extant *hisA* and *hisF* genes should have been the elongation of a gene half the size of the extant ones. Interestingly, HisA is the only one maintaining an almost perfect subdivision in two modules that are half the size of the entire gene and that share a high degree of sequence similarity [15].

**Figure 6:**
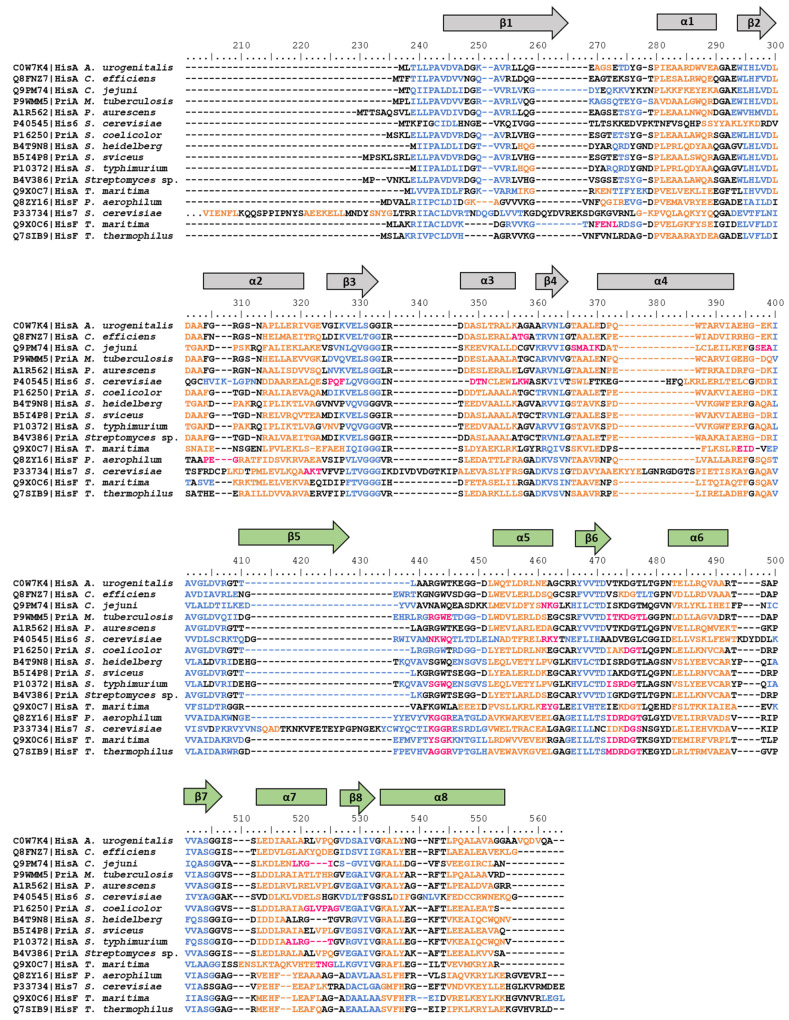
Alignment of the amino acid sequences of HisA, HisF, and relative orthologs reported in the PDB. Secondary structure elements are reported in orange (α-helices), blue (β-strands), and fuchsia (turns). α-helices and β-strands are also schematized with rectangles and arrows, respectively; the elongation event is depicted with the two halves of the proteins colored in grey and green.

In the present work, for those organisms for which HisA and/or HisF (or their relative orthologs, as PriA for *Streptomyces* spp. and *M. tuberculosis*, and His6 and His7 for *Saccharomyces cerevisiae*) 3D structures were available in the PDB (see Materials and Methods), the secondary structure was investigated, highlighting the conservation of the α-helix and β-strand repetitions among members of different taxonomic groups (Figure 6).

All these findings support the idea that the reaction specificities of current (βα)_8_-barrel enzymes evolved from an ancestral enzyme with low substrate specificity, which appeared before LUCA. Subsequently, this enzyme must have undergone several evolutionary divergence events, leading to the high variety of TIM-barrel proteins currently present. An important corollary of this idea is that (βα)_8_-barrel enzymes might be engineered to acquire new activities [19].

### 3.2. *hisB*

While a huge amount of information regarding their early evolution is available for *hisA* and *hisF*, for the histidine biosynthetic genes *hisB* and *hisD*, only a few data are available, and they mainly concern the crystal structures of their gene products. These analyses allowed the possibility of a gene elongation event having occurred in the evolution of these genes to be suggested. In both cases, an internal sequence repeat was observed, presumably deriving from an in-tandem gene duplication of a starting module followed by the fusion of the derived copies.

The *hisB* gene codes for an imidazole-glycerol-phosphate dehydratase (IGPD). It catalyzes the sixth step of histidine biosynthetic pathway, the Mn^2+^-dependent dehydration of imidazole-glycerol-phosphate to imidazole-acetyl-phosphate (Figure 2) [17,47]. IGPDs from fungi [48,49], plants [50,51], Archaea, and some Bacteria are monofunctional [52]. Other bacterial genomes harbor bifunctional genes, in which IGPD coding gene is fused to the histidinol-phosphate phosphatase one, the penultimate enzyme of histidine biosynthesis [17]. Previous structures of IGPDs available in the PDB, belonging to all the three cell lineages, show that IGPD is a homo 24-mer with, correspondingly, 24 active sites, each formed by residues from three adjacent subunits [53], and that it presents two manganese ions bound at each of the catalytic centers [52]. This structure and other biochemical data reveal that, unlike other enzymes in which Mn^2+^ can be exchanged with Mg^2+^ or Zn^2+^ with little effect on activity, IGPD has a peculiar requirement for manganese [47]; indeed, in the absence of Mn^2+^, plant and fungal IGPDs are stable but inactive trimers [52]. All the available three-dimensional structures belong to monofunctional IGPDs; thus, it is not possible to know whether the bifunctional ones are able to form the 24-mer too. IGPD is an interesting candidate for structural studies because of its unusual chemical reaction, its aggregation properties, the metal dependence, the lack of sequence similarity to proteins of known structure, and the potential as an herbicide target [52].

**Figure 7:**
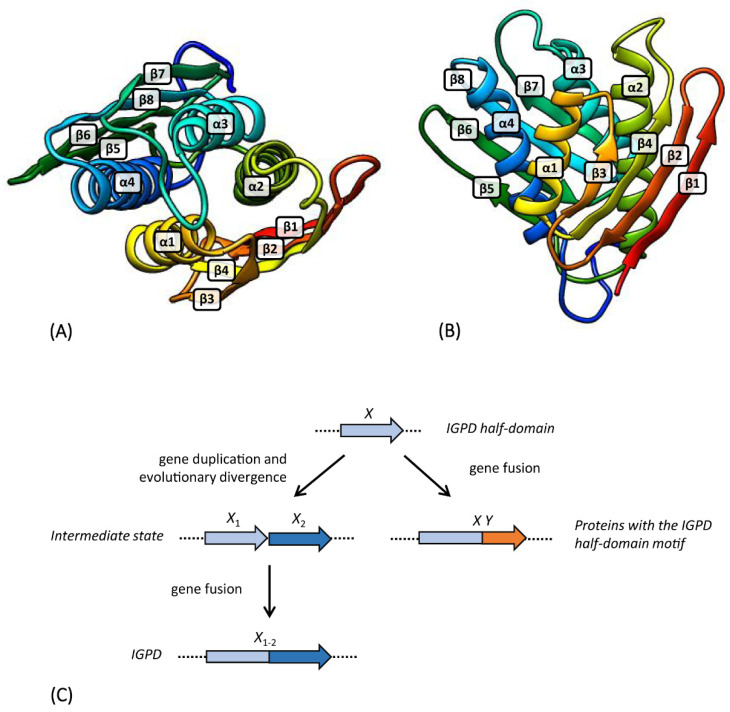
(**A**,**B**) Three-dimensional structure of IGPD domain of *E. coli* HisB. α-helices and β-strands are labeled. (**A**) Top view of IGPD; (**B**) side view. (**C**) Evolutionary model proposed to explain the origin and evolution of IGPD and proteins with subdomains with topologies identical to the IGPD half-domain.

Sinha et al. [52] analyzed the crystal structure of IGPD from the fungus *Filobasidiella neoformans*. This analysis revealed a new kind of structure in which an unusual structural motif is duplicated into a single compact domain. The IGPD polypeptide is composed of four α-helices (α1-α4), located between two four-stranded β-sheets (β1-β2-β4-β3 and β5-β6-β8-β7) (Figure 7A,B). This configuration possesses an internal repeat, in which the first β-sheet and two α-helices (β1-β2-β3-α1-β4-α2) have an identical topology to the second β-sheet and two α-helices (β5-β6-β7-α3-β8-α4). They observed that the structural repeat matches an internal sequence repeat; in particular, the amino acid sequences of the two halves of *F. neoformans* IGPD are 19% identical. Each half-domain includes a specific motif, Asx-Xaa-His-His-Xaa-Xaa-Glu [(D/N)XHHXXE], that is highly conserved in IGPD sequences. A subsequent experiment performed by Glynn et al. [54] showed that the structure of the monomer of *Arabidopsis thaliana* IGPD closely resembles that of the *F. neoformans* enzyme, with the two halves of the molecule related by a two-fold pseudo-symmetrical axis.

**Figure 8:**
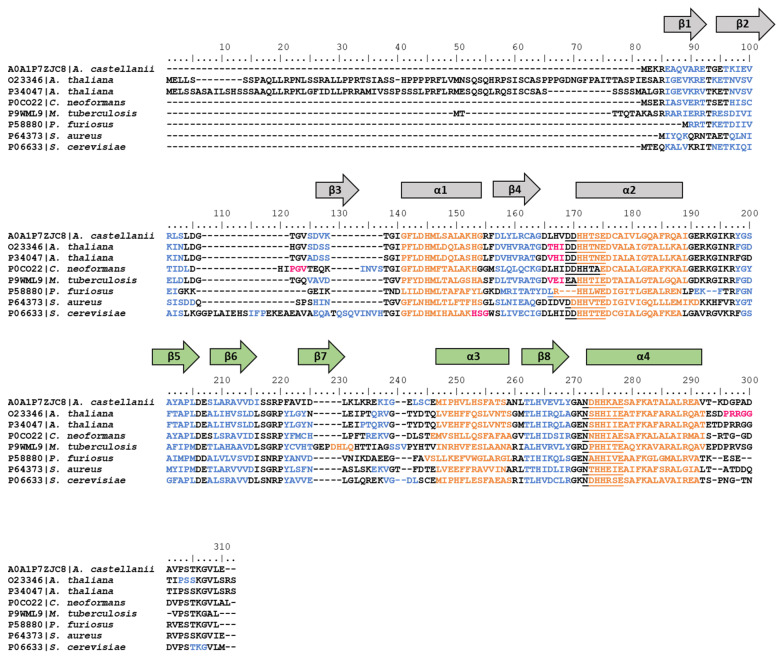
Alignment of the amino acid sequences of IGPDs reported in the PDB. Secondary structure elements are reported in orange (α-helices), blue (β-strands), and fuchsia (turns). The (D/N)XHHXXE motifs are underlined. α-helices and β-strands are also schematized with rectangles and arrows, respectively; the elongation event is depicted with the two halves of the proteins colored in grey and green.

In Sinha et al. [52], a multiple-sequence alignment of IGPD from 56 organisms was performed. In the present work, 81 IGPD sequences from Archaea, 359 from Bacteria, and 95 from Eukarya were aligned, for a total of 535 IGPD sequences. The repetition of the (D/N)XHHXXE motif between each half-domain of IGPD was strongly conserved among all the analyzed sequences, and this motif also proved to be highly conserved between the IGPD of the different organisms. In particular, the HHXXE motif was present in all the analyzed sequences. On the other hand, the D/N residues appeared to be more variable; indeed, they were totally conserved in Eukarya, while in Bacteria and Archaea they were less conserved. Moreover, for those organisms with IGPD structures reported in the PDB (see Materials and Methods), the secondary structure was investigated, confirming the presence of the internal structural repetition (Figure 8). As proposed by Sinha et al. [52], and on the basis of these additional results, the repetition of sequence and structural elements strongly suggests a gene elongation event in the evolution of IGPD. The presence of this highly conserved motif in both the half-domains of all the analyzed IGPD sequences belonging to the three cell lineages suggests that the gene elongation event leading to the extant IGPDs very likely took place very early during evolution, before the divergence from LUCA.

As reported by Sinha et al. [52] and Glynn et al. [54], according to a topology search in the PDB, no other examples of proteins with folds like the IGPD have emerged. However, several proteins have subdomains with topologies identical to the IGPD half-domain; the presence of the IGPD half-domain motif in other proteins supports the hypothesis that it constitutes an initial folding unit, which evolved to produce the extant IGPD via gene elongation [52]. Thus, it is possible to hypothesize that the IGPD half-domain might represent a starter type that subsequently underwent different fates, namely a gene elongation event which led to the formation of the current complete IGPD or a fusion event with other genes, leading to the evolution of proteins possessing subdomains with the IGPD half-domain topology (Figure 7C).

### 3.3. *hisD*

The *hisD* gene encodes the enzyme L-histidinol dehydrogenase (HDH), which has a bifunctional activity catalyzing the last two steps in histidine biosynthetic pathway (Figure 2). This enzyme converts L-histidinol (HOL) to L-histidine through a L-histidinaldehyde (HAL) intermediate. The bifunctional enzymatic activity of the *hisD* gene product seems to be a universal property [17], and the sequence of HisD has been well conserved during evolution from bacteria to fungi and plants [25,55]. HDH is a homodimeric enzyme containing two metal binding sites per enzyme dimer, which normally bind Zn^2+^, but which may also bind Mn^2+^ and still retain enzyme function [56].

**Figure 9:**
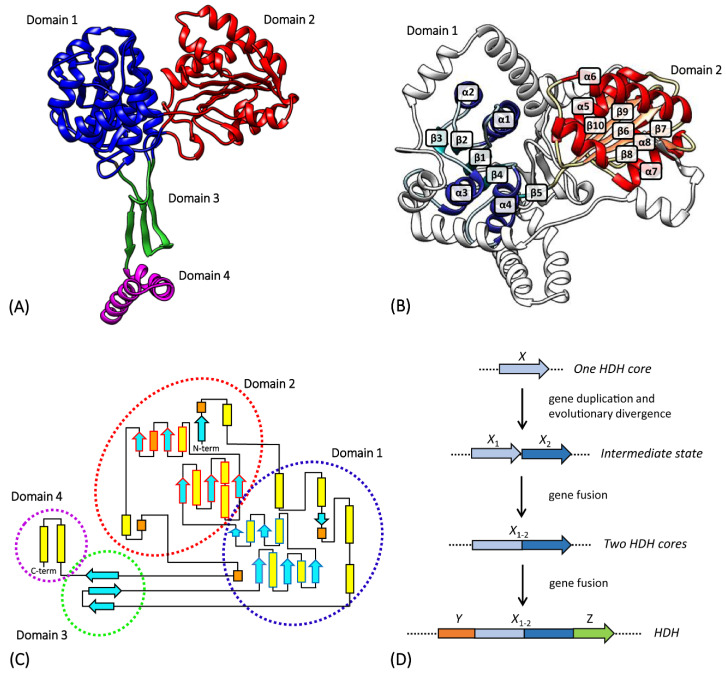
(**A**) Side view of the three-dimensional structure of the *E. coli* HisD monomer. The different domains are highlighted with different colors: Domain 1 in blue, Domain 2 in red, Domain 3 in green, and Domain 4 in violet. (**B**) Top view of *E. coli* HisD monomer: the elements of the two HDH cores are colored in blue (Domain 1) and red (Domain 2). α-helices and β-strands of the cores are labeled. (**C**) Topology diagram. Secondary structure elements are colored in yellow (α-helices), orange (3_10_ helices), and light blue (β-strands). The four domains are circled with the corresponding colors used in (**A**). The cores of the two Domains are highlighted with the secondary structure element outlines colored in blue (in Domain 1) and red (in Domain 2). Diagram adapted from Barbosa et al. [18] and Ruszkowski and Dauter [58]. (**D**) Evolutionary model proposed to explain the origin and evolution of HDH.

Barbosa et al. [18], through the analysis of the crystal structure of *E. coli* HisD, observed a possible early event of gene elongation. The HisD monomer consists of a globule and a tail. The globule is made of two larger domains (1 and 2) and the extending L-shaped tail of two smaller domains (3 and 4) (Figure 9A). Domain 1 and Domain 2 display an α/β/α topology and they both contain a core with an incomplete Rossmann fold (with only five strand–helix hairpins, instead of six) (Figure 9B,C). When the two domains are superposed, their cores appear to be very similar, with only 11% amino acid sequence identity, but with a similarity value of about 41%. The resemblance of the three-dimensional structure of the two cores, the amino acid sequence similarity, and their tandem occurrence with only two residues between the end of the first and the start of the second suggested a gene duplication event followed by a fusion, which must have occurred early in the evolution of this enzyme; subsequently, the two moieties diverged and only one of the two domains retained the ability to bind NAD^+^, whereas the other evolved to bind Zn^2+^ and the substrate [18]. Indeed, the low amino acid sequence identity could be the result of an accumulation of mutations during time, suggesting that this gene elongation event presumably took place very early during evolution. In the present work, a possible evolutionary model for the evolution of HisD is proposed: starting from a gene encoding one of the two HDH cores, this could have undergone a gene elongation event. The resulting gene composed of the two cores then fused with two other regions, which formed the N-terminal and the C-terminal parts of the protein (Figure 9D).

To the best of our knowledge, only three publications on crystal structures of HDH enzymes are available: *E. coli* HDH (*Ec*HDH) [18], *Brucella suis* HDH (*Bs*HDH) [57], and *Medicago truncatula* HDH (*Mt*HDH) [58]. As reported by Ruszkowski and Dauter [58], the sequences of plant HDHs are highly similar, and significant similarities have also been highlighted between the plant and bacteria kingdoms. Furthermore, structural analyses have shown that *Mt*HDH is similar to the two aforementioned bacterial HDH.

**Figure 10:**
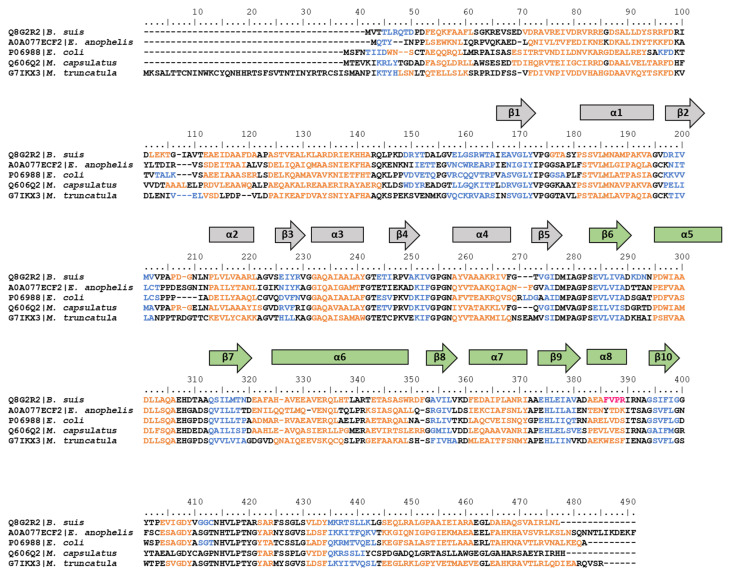
Alignment of the amino acid sequences of HDHs reported in the PDB. Secondary structure elements are reported in orange (α-helices), blue (β-strands), and fuchsia (turns). α-helices and β-strands of the two cores of the proteins are also schematized with rectangles and arrows, respectively; the elongation event is depicted with the two cores of the proteins colored in grey and green.

For those organisms with histidinol dehydrogenase structures available in the PDB (see Materials and Methods), we investigated the secondary and the three-dimensional structures. This analysis highlighted the conservation of the domain structures of the HisD monomer among different phylogenetic lineages (Figure 10). It is possible that, also in this case, the gene elongation event involving the core, as well as the fusion of genes encoding the other moieties, occurred before the appearance of LUCA.

## 4. Conclusions

As described above, the histidine biosynthetic pathway represents an excellent model for the study of molecular mechanisms that occurred during the early evolution of biosynthetic pathways. Indeed, it is known that at least three gene rearrangements shaped this route during time, i.e., gene duplication, fusion, and elongation. Concerning the third mechanism, the most documented case of gene elongation involves *hisA* and *hisF*; many studies have been performed in the last 20 years with the aim to better understand the evolutionary processes that led to the formation of the extant genes. The hypothesis for their evolution, starting from a gene half the length of the current genes that underwent a tandem event of “duplication and fusion”, is confirmed by the high sequence similarity between the two halves of the proteins, and by structural and biochemical studies. In this work, we suggest an implementation of the model, in the sense that the two extant genes could be the result of a cascade of gene elongations or gene elongation/domain shuffling events starting from an ancestor gene coding for just one (β/α) domain. In our opinion, this ancestral mini gene might represent one of the starter types, according to the definition proposed by Lazcano and Miller [44].

Similar evolutionary dynamics seem to also have occurred in the case of both *hisB* and *hisD*. Although a smaller number of studies have been conducted on these genes, structural analyses revealed the possibility of early elongation events of ancestor genes resulting in the repetition of modules, which might have been followed by the fusion of additional genes coding for other moieties (in the case of *hisD*).

**Figure 11:**
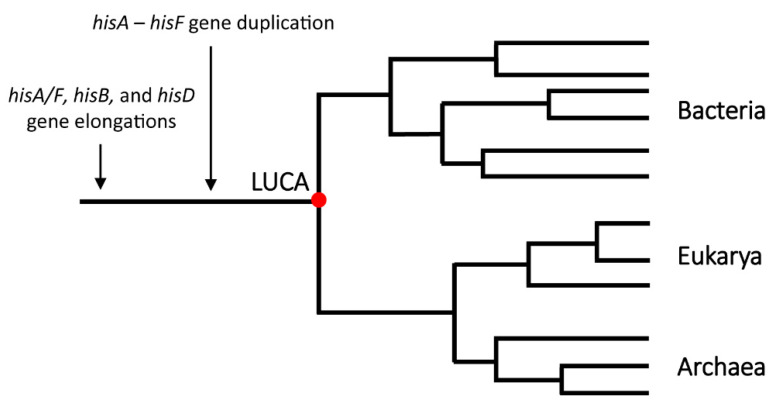
Schematic representation of the occurrence of the gene elongation events in the evolution of *his* genes throughout phylogeny.

Furthermore, since the structures of the four proteins are highly conserved in the prokaryotic and eukaryotic histidine-synthesizing (micro)organisms, it is quite possible that these gene elongation events occurred very early in the evolution of *hisA*/*hisF*, *hisB*, and *hisD* and predated the appearance of LUCA (Figure 11), which is in agreement with the idea that histidine biosynthesis is a very ancient metabolic route.

In conclusion, gene elongation events seem to have played a crucial role in the evolution of the histidine biosynthetic pathway. It is not clear whether other histidine biosynthetic genes might be the result of other gene elongation events; indeed, the analysis of the amino acid sequence of the other enzymes involved in histidine biosynthesis from archaeal and/or bacterial strains did not reveal a degree of sequence similarity sufficient to suggest the presence of internal repetitions. This was in agreement with the analysis of the bibliographic data, which, to the best of our knowledge, did not report the presence of such repetitions on the basis of the crystal structures analyses. However, by assuming that histidine biosynthesis is a very ancient pathway, it could be possible that very old gene elongation events (that can be disclosed by analyzing the structure or the degree of sequence similarity) might be obscured by the long divergence time and/or by the lower structural constraints in other His proteins. Even though this issue is beyond the scope of this work, it is also possible that other *his* genes could be the result of duplication events of genes embedded with a broad range of substrates, i.e., also involved in other metabolic pathways, and might have been recruited into the histidine biosynthetic route, in accordance with the patchwork hypothesis [42].

## Supplementary Materials

The following are available online at https://www.mdpi.com/2076-2607/8/5/732/s1, Supplementary Material S1: files of the predicted three-dimensional structures of *E. coli* HisA, HisF, HisB (IGPD) and HisD (HDH) in PDB format. Supplementary Material S2: alignment of the 535 IGPD sequences. Accession numbers of all the considered organisms are reported.
